# EMA and NICE Appraisal Processes for Cancer Drugs: Current Status and Uncertainties

**DOI:** 10.1007/s40258-018-0393-7

**Published:** 2018-05-28

**Authors:** Rumona Dickson, Angela Boland, Rui Duarte, Eleanor Kotas, Nerys Woolacott, Robert Hodgson, Rob Riemsma, Sabine Grimm, Bram Ramaekers, Manuela Joore, Nasuh Büyükkaramikli, Eva Kaltenthaler, Matt Stevenson, Abdullah Pandor, Steve Edwards, Martin Hoyle, Jonathan Shepherd, Xavier Armoiry, Miriam Brazzelli

**Affiliations:** 1 Liverpool Reviews and Implementation Group, University of Liverpool, Whelan Building, Liverpool, L69 3GB UK; 2 Centre for Reviews and Dissemination, University of York, York, UK; 3 Kleijnen Systematic Reviews, York, UK; 4 Department of Clinical Epidemiology and Medical Technology Assessment, Maastricht University Medical Center, Maastricht, The Netherlands; 5 Institute for Medical Technology Assessment, Erasmus University Rotterdam, Rotterdam, The Netherlands; 6 School of Health and Related Research, The University of Sheffield, Sheffield, UK; 7 BMJ Technology Assessment Group, BMJ, London, UK; 8 Peninsula Technology Assessment Group, University of Exeter Medical School, Exeter, UK; 9 Southampton Health Technology Assessments Centre, University of Southampton, Southampton, UK; 10 Warwick Evidence, University of Warwick, Coventry, UK; 11 Health Services Research Unit, University of Aberdeen, Aberdeen, UK

## Appraisal Processes for Cancer Drugs

The European Medicines Agency (EMA) began operating in 1995 with the aim of evaluating the clinical efficacy and safety of new medicines prior to their entry into the European Union (EU) market. In addition, the EMA ensures that the benefits of the medicines authorised for use in the EU outweigh their risks by continuing to monitor their safety after approval through their pharmacovigilance programme [1].

Recently, Davis et al. [2] reported that the majority of oncology drugs approved by the EMA between 2009 and 2013 were “…without evidence of benefit on survival or quality of life” [2]. The authors reported that, at the time of EMA assessment, significant prolongation of survival was reported for 24 out of 68 indications (35%) and there was an improvement in quality of life for seven out of 68 indications (10%). The clinical benefit of the new drugs remained uncertain for 33 out of 68 indications (49%) after a median follow-up of 5.4 years post-marketing authorisation [2].

In England, the National Institute for Health and Care Excellence (NICE) has a mandate to appraise drugs approved by the EMA in a timely fashion with a view to making recommendations regarding their routine use in the National Health Service (NHS) [3]. This mandate makes it necessary for NICE appraisals to be undertaken using the data available at the time of, or near the point of, regulatory approval. The level of evidence that informs the EMA’s conclusion of a positive benefit/risk balance therefore plays a large part in determining the level of uncertainty present in each NICE appraisal.

Evidence submissions from the sponsors of new oncology drugs to NICE are critiqued by independent Evidence Review Groups (ERGs) and subsequent recommendations for use are made by one of the four NICE appraisal committees. In this commentary, authored by representatives of the nine ERGs, we report the results of our comparison of the oncology drugs approved by the EMA between 2009 and 2013 [2] and the appraisal decisions made by NICE in the Single Technology Appraisal (STA) process. We then reflect on the newly revised Cancer Drugs Fund (CDF) [4] and highlight some of the challenges that we feel policy makers may face in the future.

## Methods

Electronic searches were conducted using the NICE guidance website [5] to identify the drugs approved by the EMA for oncology indications between 2009 and 2013. We then determined how many of these drugs had been considered as part of the NICE appraisal process and recorded the resultant decisions taken by the NICE appraisal committee. Finally, we reviewed the CDF listings [6] to identify how many of these drugs were available to the NHS through this fund.

Data extracted included the NICE appraisal committee recommendation following publication of the final appraisal determination, whether there were limitations in the recommended use of the drug and if the drug was to be provided at a discounted cost in the UK (e.g. via a patient access scheme).

## NICE Recommendations

The NICE appraisal committees can issue a number of possible recommendations in any given appraisal. The recommendations can be classified into the following categories [5]:

- recommended for use at the stated price or at a discounted price offered by the sponsor of the submission;
- recommended for optimised use, meaning that the technology is only recommended for a subgroup of the patients listed in the EMA marketing authorisation (e.g. based on factors such as disease stage or progression, or receipt of previous treatments);
- recommended for use within the new CDF;
- recommended for use only in research;
- not recommended.

A summary of the decisions taken by NICE related to the 68 cancer indications for drugs that the EMA approved between 2009 and 2013 is presented in Table 1. There are a number of interesting issues to examine from these data. NICE awarded a positive recommendation in 45 out of the 57 oncology indications (79%) that it did appraise. Of these, eight (18%) received what is known as an optimised recommendation. In addition, of the 45 positive recommendations, 37 (82%) were for oncology drugs that were recommended by NICE only when they were made available at a discounted price to the NHS. Finally, NICE did not carry out an appraisal of 11 of the drugs approved by the EMA between 2009 and 2013.

**Table 1:** NICE decisions for indications related to the cancer drugs approved by the EMA between 2009 and 2013

Outcome	Positive recommendation	Optimised recommendation	Cancer Drugs Fund	Total
Positive recommendation (at list price)	6	2	0	8
Positive recommendation at a discounted price	15	6	16	37
Not recommended				12
No NICE recommendation				11
No company submission [n = 3]				
Unable to differentiate drug and indication on the NICE website [n = 3]				
Not referred to NICE [n = 2]				
Suspended or discontinued [n = 2]				
In progress [n = 1]				
Total	21	8	16	68

Therefore, it can be seen that the NICE appraisal process is fulfilling its mandate through the application of rigorous appraisal processes that take into consideration both clinical and cost effectiveness whilst attempting to ensure that patients receive effective treatments in a timely manner.

## Referrals to the UK Cancer Drugs Fund

In an analysis of the UK CDF, Aggarwal et al. [7] (like Davis et al. [2]) reported a lack of clinical benefit for the 29 cancer drugs approved for 47 indications that could have been accessed through the CDF in January 2015. Aggarwal et al. [7] concluded that “…the majority of CDF-approved indications have been based on studies that reported minimal to no benefit in survival.”

However, since July 2016, there has been a significant change in the CDF with the responsibility for it shifting to NHS England and NICE [8]. In the new process, NICE refers a cancer drug to the CDF in situations where there is a ‘plausible potential’ that, with additional data, the drug could be recommended for routine commissioning in the NHS [9]. The drug is then available to patients through the fund and further data on effectiveness can be collected to inform a new appraisal of the cost effectiveness of the intervention at a future date, usually within 2 years of the initial referral. Up until March 2018, NICE had referred a total of 36 drugs (for 53 different indications) to the CDF. Of the oncology treatments considered in this commentary, a total of 15 drugs for 16 indications approved by NICE are being monitored through the CDF.

## Current Uncertainties

NICE has an important role as an assessor of both the clinical and cost effectiveness of oncology drugs approved for use by the EMA. However, as outlined by Woolacott et al. [10], there are methodological challenges to assessing effectiveness when the clinical evidence available to the regulators is limited and/or immature. For example, there has been an increase in the number of NICE appraisals where the only clinical effectiveness data available for consideration comes from single-arm, non-comparative studies that often have small numbers of patients and limited follow-up. Over the past year (i.e. March 2017 to March 2018), there have been 14 such submissions that the ERGs have been asked to critique. Having to make decisions based on limited data invariably leads to assumptions in economic models that consequently result in increased uncertainty in the cost-effectiveness results, which is often not appropriately reflected (i.e. not parameterised in the model, not sufficiently explored in scenario analysis). Given these complexities and the considerable uncertainty, it is not clear whether all of the positive recommendations by NICE would be justified had more definitive data been available, or whether such recommendations truly resulted in improvements in patient outcomes.

We acknowledge that, given all of these uncertainties, it is becoming more challenging for NICE and other national HTA agencies to make recommendations about the adoption of clinical- and cost-effective technologies. While all HTA agencies continue to strive to improve their appraisal processes, sponsors should be encouraged not only to collect good quality clinical evidence over the long term but also to make this information routinely available to HTA agencies. There has been a recent victory in the European Court of Justice allowing for the release of clinical data by the EMA and there are hopes that there will be further steps taken to improve the transparency of clinical data [11].

In England, NICE is working hard to ensure that cancer drugs with uncertain benefits are recommended for use only when accompanied by careful monitoring via the new CDF. The potential total benefits to patients of early access to promising new treatments will be realised more quickly than ever before. We welcome this approach as it means that, where the benefits of new cancer drugs are uncertain at the time of marketing authorisation, patient outcomes can be monitored without denying patients early access to these new treatments. Drugs in the CDF are to be reviewed usually within a period of 2 years and will then undergo further appraisal with only cost-effective drugs with proven benefits being recommended for use in the NHS. It is of note that the pathway to a CDF recommendation and design of potential data collection is currently not clearly defined. The inclusion of a formal step, in which the value of data collection is assessed compared to its cost and the cost of making the new drug available through the CDF, may aid in ensuring the efficiency of the CDF.

As researchers responsible for critiquing evidence submissions to the NICE appraisal process, we find ourselves facing increasing uncertainty:

- Uncertainty in the clinical data available as we note the increase in the number of NICE appraisals that are using data from small, single-arm studies with short-term follow-up.
- Uncertainty in the way the newly introduced CDF process will monitor the drugs approved through this mechanism and how information will flow back into the NICE appraisal process.
- Uncertainty due to potential changes in the NICE appraisal process coming into effect in April 2018 that include new roles for the NICE appraisal committees, the NICE appraisal teams and the ERGs providing critiques of the submitted evidence [12].

As regulatory authorities continue to approve cancer drugs that have uncertain benefits at the time of licensing, it increasingly becomes the responsibility of every national HTA agency to ensure that the post-marketing survival benefits of these drugs are closely monitored. Indeed, willingness to address these uncertainties must be a priority for all parties involved in HTA.

Of course, added to this, there is the uncertainty of how medicines will be licensed in the UK following departure from the EU in 2019, and how, in turn, this will affect the current NICE drug appraisal process [13].
